# Effects of a palaeolithic diet on obstructive sleep apnoea occurring in females who are overweight after menopause—a randomised controlled trial

**DOI:** 10.1038/s41366-022-01182-4

**Published:** 2022-07-25

**Authors:** Karl A. Franklin, Eva Lindberg, Johan Svensson, Christel Larsson, Bernt Lindahl, Caroline Mellberg, Carin Sahlin, Tommy Olsson, Mats Ryberg

**Affiliations:** 1 Surgical and Perioperative Sciences, Surgery, Umeå University, Umeå, Sweden; 2 Medical Sciences, Respiratory, Allergy and Sleep Research, Uppsala University, Uppsala, Sweden; 3 Department of Statistics, Umeå School of Business, Economics and Statistics, Umeå University, Umeå, Sweden; 4 Food and Nutrition and Sport Science, University of Gothenburg, Gothenburg, Sweden; 5 Public Health and Clinical Medicine, Sustainable Health, Umeå University, Umeå, Sweden; 6 Public Health and Clinical Medicine, Medicine, Umeå University, Umeå, Sweden

**Keywords:** Randomized controlled trials, Obesity

## Abstract

**Background/Objectives:**

Obesity is the main risk factor for obstructive sleep apnoea, commonly occurring in females who are overweight after menopause. We aimed to study the effect of a palaeolithic diet on sleep apnoea in females with overweight after menopause from the population.

**Methods:**

Seventy healthy, non-smoking females with a mean age of 60 years and a mean BMI of 33 kg/m^2^ were randomised to a palaeolithic diet or to a control low-fat diet according to Nordic Nutritional Recommendations, for 2 years. The apnoea-hypopnoea index was measured and daytime sleepiness was estimated during the intervention.

**Results:**

The mean apnoea-hypopnoea index at baseline was 11.6 (95% CI 8.6–14.5). The mean weight loss was 7.2 kg (95% CI 5.3–9.2 kg) in the palaeolithic diet group and 3.9 kg in the control group (95% CI 1.9–5.9 kg); *p* < 0.021 for the group difference. The reduction in weight corresponded to a reduction in the apnoea-hypopnoea index in the palaeolithic diet group (*r* = 0.38, *p* = 0.034) but not in the control group (*r* = 0.08, *p* = 0.69). The apnoea-hypopnoea index was reduced in the palaeolithic diet group when the weight was reduced by more than 8 kg. Daytime sleepiness according to the Epworth Sleepiness Scale score and the Karolinska Sleepiness Scale score was unaffected by dietary group allocation.

**Conclusions:**

A substantial decrease in body weight of 8 kg was needed to achieve a reduction in sleep apnoea in this small trial of women who are overweight after menopause. The palaeolithic diet was more effective for weight reduction than a control low-fat diet and the reduction in sleep apnoea was related to the degree of weight decrement within this diet group.

**Trial registration:**

Clinicaltrials.gov: NCT00692536.

## Introduction

Obstructive sleep apnoea is now estimated to affect almost a billion people worldwide [1] and up to 80% of women with obesity develop sleep apnoea [2]. Women after menopause run a particular risk of developing sleep apnoea. This may relate to fat mass redistribution, sex-hormone changes and age per se [3, 4]. Action to reduce weight in the population is needed to counteract poor health outcomes from obstructive sleep apnoea [5–13].

Obesity is an important risk factor for sleep apnoea and these patients are often recommended to reduce the severity of sleep apnoea by weight reduction. There are, however, mixed results regarding the resolution of sleep apnoea by dietary interventions or bariatric surgery [14–21]. The reason for this is unclear, but it may be due to the degree of weight reduction.

We and others have shown that a palaeolithic diet reduces body weight and improves metabolic factors [22–24]. The diet mimics our ancestors’ cuisine, with low carbohydrates and a high intake of monounsaturated fats and omega-3 fatty acids. To the best of our knowledge, no study has investigated the effect of a palaeolithic diet on sleep apnoea in people who are overweight from the population. This study aimed to investigate the effect of a palaeolithic diet on sleep apnoea in women who were overweight after menopause, compared with a low-fat control diet.

## Methods

**Fig. 1:**
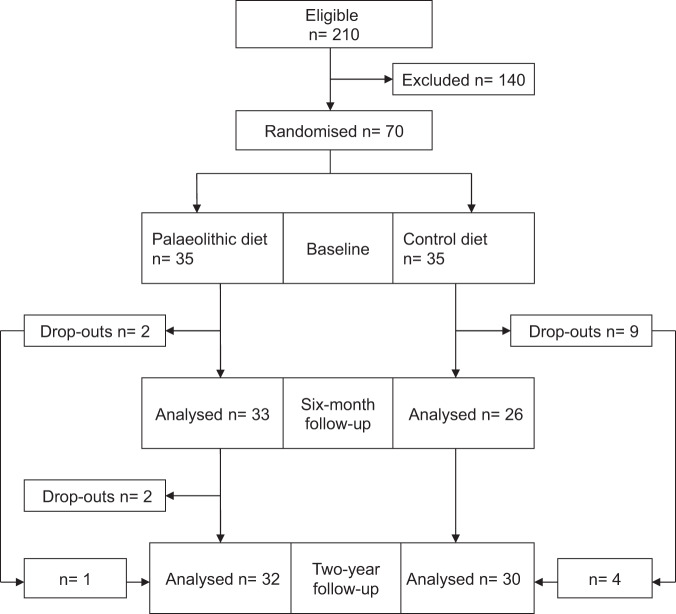
Flow chart of participants in the study. The inclusion of dropouts in the intention-to-treat analysis is noteworthy.

This is a secondary analysis from a randomised controlled trial comparing a palaeolithic diet with a low-fat control diet according to the Nordic Nutritional Recommendations, conducted in Umeå, Sweden [24]. The eligible group comprised 210 females who responded to advertisements in two local newspapers. Seventy females fulfilled the inclusion criteria of being postmenopausal, non-smoking, body mass index >27 kg/m^2^, healthy and free from medication, except for three women with well-controlled hypertension treated with an angiotensin-converting enzyme inhibitor. Investigations were recorded at baseline, after 6 months and after 2 years and included sleep apnoea recordings, subjective sleepiness scales, weight and height (Fig. 1). All the participants were invited to a follow-up, intention-to-treat analysis, regardless of whether they had continued the allocated diet. The participants were recruited from 4 September 2007 to 29 February 2008. Follow-up investigations were made from 4 October 2009 to 22 June 2010.

### Ethical approval

The Regional Ethical Review Board at Umeå University approved the study protocols (no. 07-034 M), in accordance with the Helsinki Declaration, and all the patients gave their written informed consent.

### Randomisation and masking

The included females were put in order according to their body mass index and randomised in blocks of four with an equal probability of being randomised to the palaeolithic diet (*n* = 35) or to the low-fat control diet (*n* = 35). The randomisation sequence was created by a statistician, using statistical software (IBM SPSS v. 19, Chicago, IL). All study personnel except the dieticians were blinded to dietary allocation.

### Outcome measurements

The primary outcome of the trial was a change in fat mass over a period of 2 years [24]. Secondary outcomes included the apnoea-hypopnoea index and daytime sleepiness.

### Procedures

#### Dietary intervention

The palaeolithic diet was based on lean meat, fish, eggs, vegetables, fruits, berries and nuts. Additional fat sources were avocado, rapeseed oil and olive oil. Dairy products, cereals, added salt, refined fats and sugar were excluded. The diet aimed at 30% of energy intake from protein, 40% of energy intake from fat, with a recommended high intake of mono- and polyunsaturated fatty acids, and 30% of energy intake from carbohydrates [24]. The control diet according to the official Nordic Nutritional Recommendations was based on low-fat and high-fibre products, aiming at a daily intake of 15% energy intake from protein, 25–30% energy intake from fat and 55–60% energy intake from carbohydrates [25].

One dietician per dietary group held 12 group sessions. Four cooking classes and four follow-up sessions were held during the first 6 months, followed by group meetings at 6, 12, 18 and 24 months. Participants were recommended to eat the advised food at three main meals and two snacks a day. Food intake was ad libitum for both diets, meaning that women could eat as much as they liked, without restriction. Recipes, written instructions and suggestions of food for breakfast, lunch and dinner were given during the 12 group sessions. The group sessions consisted of information on how to prepare and cook meals and dishes in the intervention diet. The sessions also included information about dietary effects on health, body weight and how to maintain behavioural changes. The group session on behavioural change was devoted to a discussion of different aspects of motivation, including group discussions of benefits and difficulties changing diet. Adherence to the diet intervention was monitored using self-reported 4-day food records at study start, monthly during the first 6 months and at 9, 12, 18 and 24 months. Participants were instructed to estimate the amount of food eaten from coloured food-portion photographs and household measuring utensils. Adherence to the different diets was assessed using the Dietist XP nutritional analysis package (version 3.0, Kost och Näringsdata AB, Bromma, Sweden), which converted the food intake to estimates of energy intake and nutrients. Adherence to protein intake was measured by nitrogen excretion in urine at baseline and after 6 and 24 months [24]. Body weight was measured at baseline and after 6, 12, 18 and 24 months.

### Sleep apnoea recordings

The apnoea-hypopnoea index was measured with overnight sleep apnoea recordings (Embletta X10, Natus Medical Inc., CA, USA). It included continuous recordings of airflow using a nasal flow pressure sensor, thoracic and abdominal respiratory effort (XactTrace respiratory effort belts), finger pulse oximetry (Nonin Oximeter XPOD) and a body position sensor.

An obstructive apnoea was defined as a drop in airflow of at least 90% of the pre-event baseline for at least 10 s with continuing abdominal and thoracic movements, according to the American Academy of Sleep Medicine [26]. An obstructive hypopnoea was defined as a 50% reduction in airflow for at least 10 s compared with baseline, accompanied by abdominal and thoracic movements in combination with an oxygen desaturation of 3% or more. A central apnoea was scored as absent inspiratory effort throughout the entire period of airflow absence lasting 10 s or more [26]. All recordings were scored manually by one of the authors (CS) and the duration of sleep was estimated from the recordings.

#### Daytime sleepiness scales

Subjective sleepiness over a longer period was measured using the Epworth Sleepiness Scale, with a summary of answers rated from 0 to 3 to eight questions on sleepiness during the daytime, leading to a summary score of 0–24 [27]. The Karolinska Sleepiness Scale that assesses immediate sleepiness on a scale from 1 to 9 was measured at awakening in the morning, at 3 pm and at 10 pm [28].

### Statistical methods

The sample size needed was estimated at 30 females in each arm to detect a significant difference in fat mass (*p* < 0.05) with a power of 80% [24]. In a post-hoc analysis, it was estimated that 65 patients in each group would be needed to detect a mean (SD) apnoea-hypopnoea index difference of 5 [10] units with a power of 80% and a significance level of 5%.

Baseline characteristics were presented as means and 95% confidence intervals. The differences between baseline and outcome were analysed with Student’s *t* test when comparing the diet groups. Model evaluations were carried out with residual analysis. The Mann–Whitney U-test was used when analysing differences in the apnoea-hypopnoea index due to outlier problems. All the tests were two sided. Pearson’s correlation coefficient was used to measure the linear correlation between the change in weight and body mass index and the change in apnoea-hypopnoea index. All the analyses were performed on an intention-to-treat basis. They included patients with low adherence to the diet intervention and all the patients were analysed with respect to randomisation. Patients who dropped out were also invited to a follow-up. A complete case analysis was performed, and missing data were assumed to be missing at random. A significance level of 0.05 was used. SPSS Statistics for Windows, Version 26.0, Armonk, NY: IBM Corp was used for statistical analysis.

## Results

The mean apnoea-hypopnoea index at baseline was 11.6 (95% CI 8.6–14.5, range 0.3–73.8) among the 70 included females and 70% had an apnoea-hypopnoea index of 5 or more. No woman had central sleep apnoea. Two women with previously diagnosed sleep apnoea were treated with continuous positive airway pressure (CPAP), one in each dietary group, and they were investigated after three nights without treatment. The baseline characteristics did not differ between the diet groups (Table 1). Four females in the palaeolithic group and nine females in the control group quit further participation during the study period. Thirty-two females randomised to the palaeolithic diet and 30 females in the control group were investigated at follow-up after 2 years. The intention-to-treat analysis included five females who had stopped following the dietary recommendations during the study period, four in the palaeolithic group and one in the control group (Fig. 1).

**Table 1:** Baseline characteristics.

	All women *n* = 70	Palaeolithic diet group *n* = 35	Control low-fat diet group *n* = 35	*p* value
Age, years	60 (95% CI 58–61) (range 49–71)	60 (95% CI 58–61) (range 52–69)	60 (95% CI 58–62) (range 49–71)	0.637
Weight, kg	86.7 (95% CI 84.2–89.2) (range 67.0–114.3)	87.0 (95% CI 83.6–90.5) (range 67.0–114.3)	86.8 (95% CI 83.6–90.1) (range 71.7–108.9)	0.940
Body mass index, kg/m^2^	32.5 (95% CI 31.7–33.3) (range 27.3–44.6)	32.7 (95% CI 31.5–33.9) (range 27.3–44.6)	32.5 (95% CI 31.4–33.7) (range 28.3–40.0)	0.803
Apnoea-hypopnoea index	11.6 (95% CI 8.6–14.5) (range 0.3–73.8)	11.1 (95% CI 8.0–14.1) (range 0.9–32.3)	11.9 (95% CI 7.5–16.5) (range 0.3–73.8)	0.774

The data are presented as the means, 95% confidence intervals and range.

In the palaeolithic diet group, body weight decreased by a mean of 7.2 (95% CI 5.3–9.2) kg, *p* < 0.001, from baseline to follow-up after 2 years, and the corresponding weight reduction was 3.9 kg (95% CI 1.9–5.9 kg) in the control group The between-group difference in weight was 3.4 (95% CI 0.5–6.2) kg, (*p* = 0.021). There was no between-group difference in the apnoea-hypopnoea index at 2 years (Table 2). The effect on outcome remained in a sub-analysis of women with sleep apnoea (apnoea-hypopnoea index > 5) at baseline (Table 3).

**Table 2:** Longitudinal analysis of outcome variables at 2 years in women with overweight after menopause.

	Palaeolithic diet *n* = 32		Control diet *n* = 30		Between-group difference	*p* value
	Baseline	Two years	Baseline	Two years	Baseline to two years	
Weight, kg	86.2. (82.7–89.8)	79.0 (74.9–83.1)	85.3 (81.7–89.0)	81.4 (77.1–85.6)	−3.4 (−6.2–−0.5)	0.021
AHI, events/h	11.8 (7.5–16.0)	12.3 (7.1–17.5)	12.7 (8.3–17.1)	13.9 (8.6–19.3)	−0.6 (−5.4–4.2)	0.807
ODI, events/h	15.0 (10.3–19.6)	14.8 (9.5–20.0)	18.8 (14.1–23.5)	19.1 (13.8–24.4)	−0.5 (−5.7–4.7)	0.845
Nocturnal hypoxia, SaO2 < 90%, minutes	4.7 (1.3–8.2)	6.4 (−2.9–15.6)	7.0 (3.5–10.5)	14.9 (5.5–24.3)	−6.2 (−17.7–5.3)	0.285
Sleep time,minutes	437 (420–454)	411 (388–435)	436 (418–454)	439 (414–463)	−28.8 (−63.8–6.2)	0.105
ESS	6.5 (5.3–7.8)	6.2 (4.9–7.6)	8.0 (6.7–9.3)	8.2 (6.8–9.6)	−0.4 (−1.8–1.0)	0.562
KSS on awakening	4.6 (3.8–5.5)	4.8 (4.0–5.6)	5.5 (4.6–6.4)	5.2 (4.4–6.0)	0.5 (−0.5–1.6)	0.295
KSS at 3 pm	4.0 (3.5–4.6)	3.9 (3.2–4.6)	4.7 (4.2–5.3)	4.4 (3.7–5.0)	0.1 (−0.9–1.1)	0.848
KSS at 10 pm	6.9 (6.2–7.5)	6.5 (5.9–7.8)	6.5 (5.9–7.1)	6.5 (5.9–7.1)	−0.4 (−1.4–0.6)	0.418
Supine sleep, %	27 (19–36)	29 (20–38)	32 (23–40)	33 (22–43)	−0.5 (−9.9–10.9)	0.924

The data are presented as means and 95% confidence intervals.

*AHI* apnoea-hypopnoea index, *ESS* Epworth Sleepiness Scale, *KSS* Karolinska Sleepiness Scale.

**Table 3:** Longitudinal analysis of outcome variables at 2 years in women with apnoea-hypopnoea index >5 at baseline.

	Palaeolithic diet *n* = 25		Control diet *n* = 24		Between-group difference	*p* value
	Baseline	Two years	Baseline	Two years	Baseline to two years	
Weight, kg	86.8 (82.7–91.0)	80.0 (75.2–84.7)	87.3 (82.9–91.6)	84.1(79.5–89.4)	−4.0 (−7.2–−0.82)	0.015
AHI, events/h	15.0 (10.0–20.0)	15.1 (8.7–21.4)	16.5 (11.3–21.7	18.2 (11.5–24.8)	−1.6 (−8.1–4.9)	0.622
ODI, events/h	17.4 (12.1–22.7)	17.1 (11.1–23.1)	23.3 (17.7-28.8)	24.3 (18.0–30.6)	−1.3 (−8.1–5.5)	0.696
Nocturnal hypoxia, SaO2 < 90%, minutes	5.8 (1.7–10.0)	7.8 (−3.6–19.3)	9.2 (4.9–13.5)	19.5 (7.5–31.5)	−8.3 (−23.1–6.5)	0.263
Sleep time, hours	443 (422–464)	410 (380–440)	435 (412–457)	428 (397–459)	−26.8 (−69.1–15.6)	0.209
ESS	6.2 (4.8–7.6)	6.0 (4.4–7.6)	8.8 (7.2–10.2)	8.9 (7.1–10.6)	−0.4 (−2.2–1.5)	0.710
KSS on awakening	4.9 (3.9–5.8)	5.1 (4.1–6.0)	5.8 (4.9–6.7)	5.4 (4.5–6.3)	0.5 (−0.7–1.8)	0.395
KSS at 3 pm	4.2 (3.5–4.9)	3.9 (3.2–4.6)	4.7 (4.1–5.4)	4.8 (4.1–5.6)	−0.4 (−1.6–0.8)	0.522
KSS at 10 pm	6.7 (5.9–7.5)	6.5 (5.8–7.2)	6.5 (5.7–7.3)	6.9 (6.1–7.6)	−0.5 (−1.8–0.7)	0.371
Supine sleep, %	29.6 (20.8–38.5)	32.9 (22.6–43.2)	26.2 (16.9–35.4)	30.0 (19.2–40.8)	−0.5 (−12.6–12.7)	0.930

The data are presented as means and 95% confidence intervals.

*AHI* apnoea-hypopnoea index, *ESS* Epworth Sleepiness Scale, *KSS* Karolinska Sleepiness Scale.

**Fig. 2:**
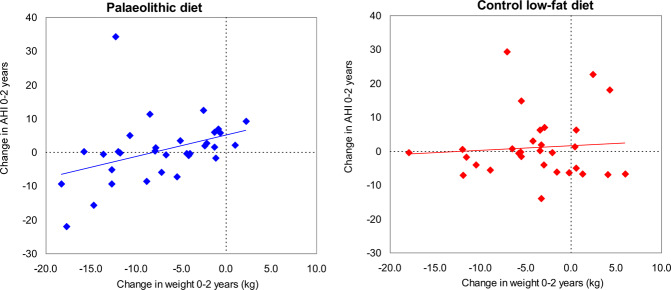
The association between weight loss in kg and change in the apnoea-hypopnoea index (AHI) in the two dietary groups. A reduction in weight corresponded to a reduction in the apnoea-hypopnoea index in the palaeolithic diet group (*r* = 0.38, *p* = 0.034) but not in the control low-fat diet group (*r* = 0.08, *p* = 0.69). A decrease in the apnoea-hypopnoea index was mainly present in individuals with a weight reduction of more than 8 kg in the palaeolithic diet group.

**Fig. 3:**
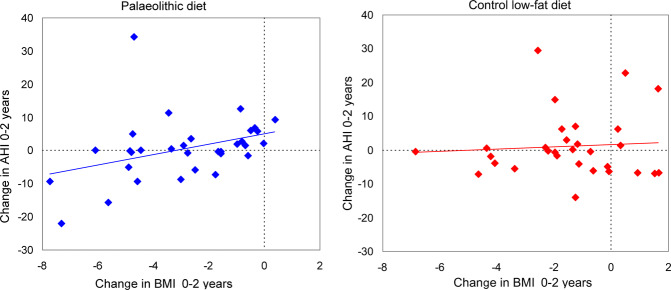
The association between change in body mass index and change in the apnoea-hypopnoea index (AHI) in the two dietary groups. A reduction in body mass index corresponded to a reduction in the apnoea-hypopnoea index in the palaeolithic diet group (*r* = 0.137, *p* = 0.037) but not in the control low-fat diet group (*r* = 0.005, *p* = 0.70).

**Fig. 4:**
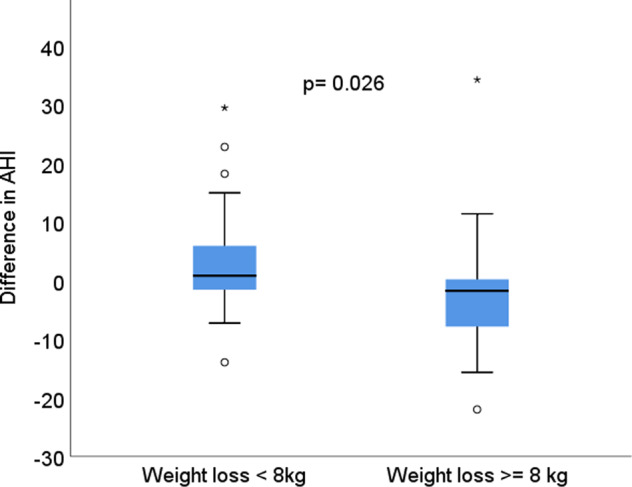
Change in apnoea-hypopnoea index (AHI) and 8 kg weight loss. The box plot illustrates the difference in the change in AHI between participants who lost ≥8 kg vs. <8 kg from baseline to follow-up after 2 years regardless of dietary intervention.

The reduction in weight corresponded to a reduction in the apnoea-hypopnoea index in the palaeolithic group (*r* = 0.38, *p* = 0.034) but not in the control group (*r* = 0.08, *p* = 0.69) (Fig. 2). The correlation between the change in body mass index and the change in the apnoea-hypopnoea index was also significant in the palaeolithic group (*r* = 0.137, *p* = 0.03) but not in the control group (*r* = 0.005, *p* = 0.70) (Fig. 3). A reduction in the apnoea-hypopnoea index after 2 years was observed in females in the palaeolithic group and in the control low-fat group, who lost more than 8 kg, *p* = 0.026 (Fig. 4). About 40% of the women in the palaeolithic group and 20% in the control group had lost more than 8 kg at follow-up.

Daytime sleepiness using the Epworth Sleepiness Scale score and the Karolinska Sleepiness Scale score on awakening, at 3 pm and at 10 pm did not change significantly from baseline to follow-up after 2 years in the palaeolithic diet group, without any difference in any score between the two randomised groups (Table 2).

## Discussion

Here, we show that the reduction in weight in the palaeolithic diet group correlated with a reduction in the apnoea-hypopnoea index. This was linked to high adherence to the palaeolithic diet for 2 years, with a substantial weight reduction in women who were overweight after menopause, compared with a low-fat diet. A reduction in sleep apnoea occurred in women with a pronounced weight loss >8 kg, regardless of the diet group. This supports the finding that, the more weight that is lost, the greater the reduction in the apnoea-hypopnea index, found in previous trials [14–16, 18].

Despite significant weight reductions, there was no significant change in the mean apnoea-hypopnea index in either diet group after 2 years. The fact that the women were 2 years older at follow-up could explain the lack of effect on the apnoea-hypopnoea index. It is known that sleep apnoea worsens with age, especially in women in the menopausal transition [2, 29]. This further underlines the importance of weight reduction in women in relation to menopause.

The weight reduction was more pronounced in the palaeolithic group and 40% of them had a weight reduction of over 8 kg vs. 20% in the control group. We found a significant, albeit weak, dose-response relationship between weight loss and improvements in the apnoea-hypopnoea index in the palaeolithic diet group. One potential mechanism may be that palaeolithic diets, compared with low-fat diets, in previous randomised controlled trials have shown greater improvements in components of the metabolic syndrome, notably waist circumference and triglyceride levels [23] and earlier studies have found close relationships between the metabolic syndrome and the apnoea-hypopnoea index [30]. A palaeolithic diet can therefore be recommended to women who are overweight after menopause, with the understanding that it will only affect the severity of sleep apnoea if weight is reduced by more than 8 kg.

The mean apnoea-hypopnoea index at baseline was 11.6 and 70% of the present women previously had unrecognised obstructive sleep apnoea. This prevalence is well in line with earlier population-based studies [2, 31] and thus constitutes a group of women running a major risk of sleep apnoea-related metabolic disorders, cardiovascular events and mortality [5–13, 32].

A systematic review from 2013 concluded that lifestyle and dietary interventions improved obstructive sleep apnoea parameters but not sufficiently to normalise them [33]. A recent study also found that interventions combining physical activity and eating behaviour reduced the severity of sleep apnoea but was not curative and the problem of how to increase the effectiveness of lifestyle modifications remains [34]. The American Thoracic Society recommends “that clinicians regularly assess weight and incorporate weight management strategies that are tailored to individual patient preferences into the routine treatment of adult patients with obstructive sleep apnoea who are overweight or obese” [35]. This statement is based on a review of weight reduction trials among patients with overweight and sleep apnoea. Effects on apnoea frequency have been observed in studies using a very low-calorie diet [14, 15], energy restriction [36, 37], bariatric surgery [21] and pharmacological treatment [38, 39]. The study with the greatest weight reduction reported the largest reduction in the apnoea-hypopnoea index [14]. In contrast, a study reporting a mean weight loss of 1.7 kg with the Mediterranean diet had no effect on sleep apnoea [17]. This is in line with our key finding, i.e. a substantial weight loss is needed to achieve a pronounced reduction in the apnoea-hypopnoea index. However, in a recent trial, Georgoulis et al. reported that the Mediterranean diet and Mediterranean lifestyle interventions, in addition to CPAP treatment, reduce the severity of obstructive sleep apnoea, regardless of CPAP and weight loss, including cardiometabolic benefits [19, 20]. This diet has some similarities with the palaeolithic diet and future studies of diets of these types in patients with sleep apnoea are warranted.

The Sleep AHEAD study presented data that support our results [16]. This study included patients with obesity and type 2 diabetes, with or without sleep apnoea at study start. Half the patients were given a very low-calorie diet during the first 4 months, plus moderate to intense exercise for 3 h a week, with the individual goal of losing at least 7% of their initial body weight. The study subjects lost a mean of 10 kg and their apnoea-hypopnoea index was reduced by 10 units per hour of sleep after 12 months vs. controls. The initial apnoea-hypopnoea index and weight reduction were the strongest predictors of a reduction in sleep apnoea. Future lifestyle interventions in females after menopause, running an increased risk of obstructive sleep apnoea because of overweight, in combination with an increased cardiometabolic risk, should focus on profound weight reduction, as there may be an individual threshold effect for effects on sleep apnoea, in line with what has recently been suggested for the remission of type 2 diabetes [40].

Excessive daytime sleepiness is suggested as the most important symptom of sleep apnoea. One review found 15 articles reporting large improvements in daytime sleepiness after bariatric surgery and 27 studies that reported a moderate improvement after non-surgical weight reduction, with a non-linear association between weight loss and change in daytime sleepiness [8]. However, only a fraction of the women and men from the population with sleep apnoea report daytime sleepiness [41] and there has been a lack of any clear relationship between daytime sleepiness and sleep apnoea among women in the population [42]. It is thus possible that daytime sleepiness in women with overweigh after menopause is primarily due to factors other than sleep apnoea. This may explain why no effect was found between weight reduction and daytime sleepiness in the present trial.

One limitation is the small sample size and the large variability in the apnoea-hypopnea index, as women both with and without sleep apnoea at baseline were included. Another limitation is the use of simplified sleep apnoea recordings instead of polysomnography including an EEG for sleep scoring. We did not control for abstinence from alcohol and caffeine before the sleep apnoea recordings, which could have affected the results. The high adherence to the palaeolithic diet, with a low drop-out rate from the study over a period of 2 years, is a strength in the present study. Further studies with large sample sizes are needed before a palaeolithic diet can be recommended to people with obstructive sleep apnoea.

## Conclusions

A substantial decrease in body weight of 8 kg was needed to achieve a reduction in sleep apnoea in women who are overweight after menopause. The palaeolithic diet was more effective for weight reduction than a control low-fat diet and the reduction in sleep apnoea was related to the degree of weight decrement within this diet group. The study sample size was limited and further studies are warranted.

## Data Availability

All data that support the findings are available on request to the corresponding authors within reason. Material and correspondence requests should be made to the corresponding author.
